# The cardiovascular safety of GLP-1 receptor agonists: From glucose control to cardiometabolic protection

**DOI:** 10.34172/jcvtr.025.33793

**Published:** 2025-12-17

**Authors:** Samad Ghaffari, Seyedeh-Tarlan Mirzohreh, Neda Roshanravan, Faezeh Tarighat

**Affiliations:** Cardiovascular Research Center, Tabriz University of Medical Sciences, Tabriz, Iran

## Introduction

 Recent advances have transformed glucagon-like peptide-1 receptor agonists (GLP-1 RAs) from glucose-lowering drugs into comprehensive cardiometabolic agents with broad cardiovascular, renal, and metabolic benefits. The most striking new evidence comes from large-scale outcome trials demonstrating that GLP-1 RAs reduce major adverse cardiovascular events (MACE) not only in patients with type 2 diabetes mellitus (T2DM) but also in non-diabetic individuals with obesity and established atherosclerotic cardiovascular disease (ASCVD).^1^

 The SELECT trial showed that once-weekly Semaglutide 2.4 mg reduced cardiovascular events by about 20% in overweight or obese patients without diabetes, marking the first demonstration of cardioprotection independent of glycemic control.^1^ Similarly, the FLOW trial confirmed that Semaglutide slowed kidney function decline and reduced kidney failure and cardiovascular death in patients with T2DM and chronic kidney disease, leading to regulatory approval for renal risk reduction.^2^

 Moreover, meta-analyses encompassing over 67,000 participants across ten major trials have confirmed that GLP-1 RAs significantly lower MACE by approximately 13% (OR 0.87, 95% CI 0.81-0.93), with parallel reductions of ~14% in cardiovascular and 13% in all-cause mortality among high-risk patients with T2DM.^3^ In post-percutaneous coronary intervention cohorts, GLP-1 RA therapy markedly reduced recurrent MACE and slowed coronary lesion progression, including in-stent restenosis.^4^ Emerging dual incretin agonists, notably tirzepatide, have demonstrated superior efficacy over established GLP-1 RAs in mitigating composite cardiovascular outcomes and mortality in patients with ischemic heart disease.^5^ Beyond diabetes, recent meta-analyses in non-diabetic populations similarly indicate consistent reductions in cardiovascular events with agents such as Liraglutide, Semaglutide, Dulaglutide, and Tirzepatide.^6^

 GLP-1 RAs have also shown promising results in heart failure with preserved ejection fraction (HFpEF); the STEP-HFpEF trial demonstrated that Semaglutide improved symptoms, exercise capacity, inflammatory markers, and quality of life, suggesting disease-modifying potential in obesity-related HFpEF.^7^

 Together, these data have repositioned GLP-1 RAs as cornerstone therapies for the integrated management of cardiometabolic disease. Beyond traditional diabetes care, they are now recognized as agents that lower atherosclerotic risk, promote weight loss, protect renal function, and improve heart failure symptoms. The emergence of newer dual and triple incretin agonists, such as Tirzepatide (GIP/GLP-1) and retatrutide (GIP/GLP-1/glucagon), as well as nonpeptide oral GLP-1 mimetics like Orforglipron, further expands the therapeutic horizon.^8^

 The mechanism of action of GLP-1RAs may include effects beyond glucose lowering, such as weight reduction, improved endothelial function, anti-inflammatory properties, and direct cardioprotective actions. Different studies consistently have shown GLP-1RAs act by inducing meaningful weight loss, lowering systolic blood pressure, improving lipid profiles (reducing LDL-C and triglycerides), and enhancing glycemic control. They may have anti-inflammatory effects, marked by lower C-reactive protein in treated patients.^9,10^ According to network meta-analyses, nuanced differences between agents, with some, like Tirzepatide, showing superior efficacy in weight and HbA1c reduction, while others, like Orforglipron, have a more pronounced impact on blood pressure, have been detected. This may be a great tool for a more personalized treatment plan.^11^

 Despite these advances, important knowledge gaps remain. Most cardiovascular outcome trials have focused on older, high-risk patients with established ASCVD, leaving uncertainty about the benefits of GLP-1 RAs for primary prevention or in younger populations with metabolic risk factors but no overt cardiovascular disease. While improvements in glycemic control, weight, and blood pressure contribute to outcome benefits, mediation analyses indicate that these account for only part of the risk reduction, implying additional direct vascular and anti-inflammatory effects whose mechanisms are not fully elucidated.^8^

 Data from preclinical and small human studies suggest that GLP-1 RAs may improve endothelial function, reduce oxidative stress, stabilize atherosclerotic plaques, and modulate immune and inflammatory pathways, yet consistent imaging and biomarker evidence at scale are lacking. Similarly, the effects of GLP-1 RAs on peripheral artery disease, cerebrovascular outcomes, and heart failure with reduced ejection fraction (HFrEF) remain uncertain due to underrepresentation of these phenotypes in existing trials.^12^

 Comparative effectiveness among individual GLP-1 RAs is also unresolved; apparent differences in MACE reduction may reflect trial design rather than true pharmacologic disparity, and direct head-to-head studies such as SURPASS-CVOT (Tirzepatide vs Dulaglutide) are still ongoing.^13^ Optimal combination strategies with SGLT2 inhibitors, statins, or novel cardioprotective agents remain undefined, as does the long-term safety of high-dose, long-duration therapy in non-diabetic populations.^8^

 Moreover, trials vary in design, endpoints, population inclusion, and follow-up duration. This heterogeneity complicates pooled analyses and broad clinical generalization. For instance, some FDA-mandated CVOTs with Lixisenatide and exenatide showed neutral effects on MACE, highlighting agent-specific differences in cardiovascular efficacy.^14^

 Most CV outcome data derive from patients with both T2D and high baseline CV risk. Data regarding low-risk or non-diabetic patients are starting to pile up. This limits clinical extrapolation and urges cautious interpretation when extending recommendations beyond high-risk groups.^6^ Additionally, the improvements observed in conventional cardiovascular risk factors such as blood pressure, lipid levels, and glycemic control are relatively modest, raising questions about whether these changes fully account for the reductions in cardiovascular events reported in clinical trials. Mechanistic investigations have also shown that GLP-1 receptor expression in cardiac and vascular tissues is limited, leaving uncertainty about whether the cardioprotective effects of these agents result primarily from direct receptor-mediated actions or indirectly through metabolic improvements in overall cardiovascular risk.^15^

 Finally, Safety concerns also have to be addressed. While GLP-1 RAs are safe for short- to medium-term use, their long-term safety is concerning. There may be a potential chance for rare but serious adverse events such as pancreatitis, gallbladder disease, and thyroid C-cell hyperplasia or tumors, which are detectable in trials of long duration and high sample size.^10^ The majority of evidence is derived from industry-sponsored studies, which may introduce bias despite independent event adjudication. Furthermore, high cost, limited accessibility, and health system inequities continue to impede global adoption, particularly in low- and middle-income countries, underscoring the need for broader, equitable implementation strategies alongside long-term safety monitoring.

## Conclusion

 In summary, GLP-1 receptor agonists have evolved into powerful cardiometabolic drugs capable of reducing cardiovascular events, kidney disease progression, and heart failure symptoms in both diabetic and non-diabetic populations. Yet key uncertainties persist regarding optimal patient selection, mechanistic pathways, long-term outcomes, and equitable global implementation. Addressing these gaps through mechanistic studies, head-to-head comparative trials, and inclusive real-world research will be essential to fully realize the cardiometabolic potential of this therapeutic class.

## Competing Interests

 The authors declare that the research was conducted in the absence of any commercial or financial relationships that could be construed as a potential conflict of interest. Some of the authors declare that they were members of the Editorial Board of the Journal of Cardiovascular and Thoracic Research at the time of submission. This had no influence on the peer-review process or the final editorial decision.

## Ethical Approval

 Not applicable.
